# Comparative Transcriptome Analyses Reveal the Role of Conserved Function in Electric Organ Convergence Across Electric Fishes

**DOI:** 10.3389/fgene.2019.00664

**Published:** 2019-07-18

**Authors:** Ake Liu, Funan He, Jingqi Zhou, Yangyun Zou, Zhixi Su, Xun Gu

**Affiliations:** ^1^Faculty of Biology Sciences and Technology, Changzhi University, Changzhi, China; ^2^School of Life Sciences, Fudan University, Shanghai, China; ^3^School of Public Health, Shanghai Jiao Tong University School of Medicine, Shanghai, China; ^4^Singlera Genomics Inc., Shanghai, China; ^5^Department of GDC Biology, Iowa State University, Ames, IA, United States; ^6^Fudan Human Phenome Institute, Shanghai, China

**Keywords:** electric organ, skeletal muscle, transcriptome, convergence, differentially expressed gene

## Abstract

The independent origins of multiple electric organs (EOs) of fish are fascinating examples of convergent evolution. However, comparative transcriptomics of different electric fish lineages are scarce. In this study, we found that the gene expression of EOs and skeletal muscles from three lineages (Mormyroidea, Siluriformes, and Gymnotiformes) tended to cluster together based on the species of origin, irrespective of the organ from which they are derived. A pairwise comparison of differentially expressed genes (DEGs) revealed that no less than half of shared DEGs exhibited parallel expression differentiation, indicating conserved directionality of differential expression either in or between lineages, but only a few shared DEGs were identified across all focal species. Nevertheless, the functional enrichment analysis of DEGs indicated that there were more parallel gene expression changes at the level of pathways and biological functions. Therefore, we may conclude that there is no parallel evolution of the entire transcriptomes of EOs among different lineages. Further, our results support the hypothesis that it is not different genes but conserved biological functions that play a crucial role in the convergence of complex phenotypes. This study provides insight into the genetic basis underlying the EO convergent evolution; however, more studies in different cases will be needed to demonstrate whether this pattern can be extended to other cases to derive a general rule for convergent evolution.

## Introduction

Convergent phenotype, referring to the evolution of identical or similar traits arising from different genetic and developmental pathways among populations or species, can offer extremely useful systems for studying the genetics of new adaptations (Arendt and Reznick, 2008; Christin et al., 2010; Stern, 2013). It has long been viewed as evidence for the claim that ecological circumstances can select for similar evolutionary solutions (Stern, 2013). The convergence of morphological traits has been observed frequently. Examples include repeated occurrences of eyes, echolocation in bats and toothed whales, multiple origins of eusociality in Hymenoptera, and convergence of some flower traits in plants (Fernald, 2006; Whittall et al., 2006; Kozmik et al., 2008; Preston and Hileman, 2009; Parker et al., 2013; Berens et al., 2015). Accordingly, convergent evolution is always associated with convergent molecular changes, such as protein-coding sequences (Li et al., 2010; Liu et al., 2010; Liu et al., 2012; Shen et al., 2012; Liu et al., 2014), cis-regulatory DNA elements (Tishkoff et al., 2007; Franchini et al., 2011), and gene expression (Arnegard et al., 2010; Thompson et al., 2014). Owing to the involvement of complex biochemical cascades as well as epistatic interactions, the identification of the genetic mechanisms underlying phenotypic convergence has remained elusive (Christin et al., 2010).

Electric organs (EOs) that produce electricity for the purposes of localization, communication, navigation, as well as predation and defense are evolutionary novelties that have arisen independently in at least six lineages (Gallant et al., 2014). These complex organs were found to be mainly distributed in the following lineages: marine electric rays (Torpediniformes), skates (Rajiformes), African freshwater Mormyridae and Gymnarchidae (Mormyroidea), South American knife fishes (Gymnotiformes), several catfish species (Siluriformes), and marine stargazers (Uranoscopidae) (Gallant et al., 2014; Lamanna et al., 2015). The EOs of all the above, except those from the family Apteronotidae, are derivatives of specialized myogenic tissues (Kirschbaum, 1983). It is one of the most remarkable examples of convergent evolution among vertebrates (Gallant et al., 2014; Thompson et al., 2014; Lamanna et al., 2015). Despite being distantly related, all focal species used in this study evolved EOs repeatedly from myogenic precursors and share striking similarities in biological functions (Lavoue et al., 2012; Gallant et al., 2014; Lamanna et al., 2015). Therefore, EOs provide a valuable model for understanding generalized principles underlying the phenotypic evolution of novel complex traits.

Similar traits among distant lineages have been regarded as a consequence of equal or similar evolutionary paths to achieve their goals. For instance, complex bioluminescent organs of independently originated squids were demonstrated to have massively parallel evolution of the entire transcriptomes (Pankey et al., 2014). However, the comparative transcriptomic analysis of caste phenotypes suggests that different genes and not conserved pathways and biological functions are involved in the eusocial evolution among three eusocial lineages (Berens et al., 2015). In addition, it has been hypothesized that completely distinct markers contribute to the independently evolved flatwing (Pascoal et al., 2014). The widely distributed convergent cases provide us with an opportunity to uncover generalized rules (or patterns) underlying the process of evolutionary novelty (Fernald, 2006; Whittall et al., 2006; Kozmik et al., 2008; Preston and Hileman, 2009; Parker et al., 2013; Gallant et al., 2014; Pankey et al., 2014; Berens et al., 2015).

Recently, researchers have studied the genetic basis of the function and evolution of the EOs (Novak et al., 2006; Zakon et al., 2006; Arnegard et al., 2010; Gallant et al., 2012; Gallant et al., 2014). The focus of some of these investigations was the duplication of genes coding for the voltage-gated sodium channel, which are associated with the origin of EOs. The diversification of these genes in electric fishes has been unraveled by identifying important functional substitutions across paralogs and discovering their differential patterns of expression in the EO and the skeletal muscle (SM) (Novak et al., 2006; Zakon et al., 2006; Arnegard et al., 2010; Thompson et al., 2014; Thompson et al., 2018). Several differentially expressed genes (DEGs), including transcription factors, ion channels, and sarcomeric proteins, in the SM and EO in several electric species have been identified using high-throughput genomic technologies (Gallant et al., 2012; Gallant et al., 2014; Lamanna et al., 2014; Lamanna et al., 2015; Traeger et al., 2015; Traeger et al., 2017). However, these previous studies have focused either on the gene expression differences between the SM and EO within the same species or on the patterns of expression of a few genes from several species. Therefore, such cases only provide limited insights into the constructive evolution of independently derived novelty functions.

Changes in gene expression are regarded as the basis for many of the phenotypic differences between species (Brawand et al., 2011). Hence, in this study, we reanalyzed the gene expression of two organs from different lineages (Mormyroidea, Siluriformes, and Gymnotiformes) to determine whether there is parallel evolution of the entire transcriptomes. Then, we compared the DEGs between these two organs among three lineages and conducted functional enrichment analysis. Our results indicated that different genetic mechanisms can be introduced to explain EO convergent phenotypic evolution. EOs from different lineages have different expression types, but genes with similar functions changed their expression during the evolution from SM to discharge organs. Our results point to convergent changes in gene expression evolution associated with convergent origins of functionally specific organs. Further, convergent evolution predictable solutions may be driven by not only the expression of the same gene but also the evolution of the gene expression levels with similar functions.

## Materials and Methods

### Data Collection and Transcriptome Assembling

**Figure 1:**
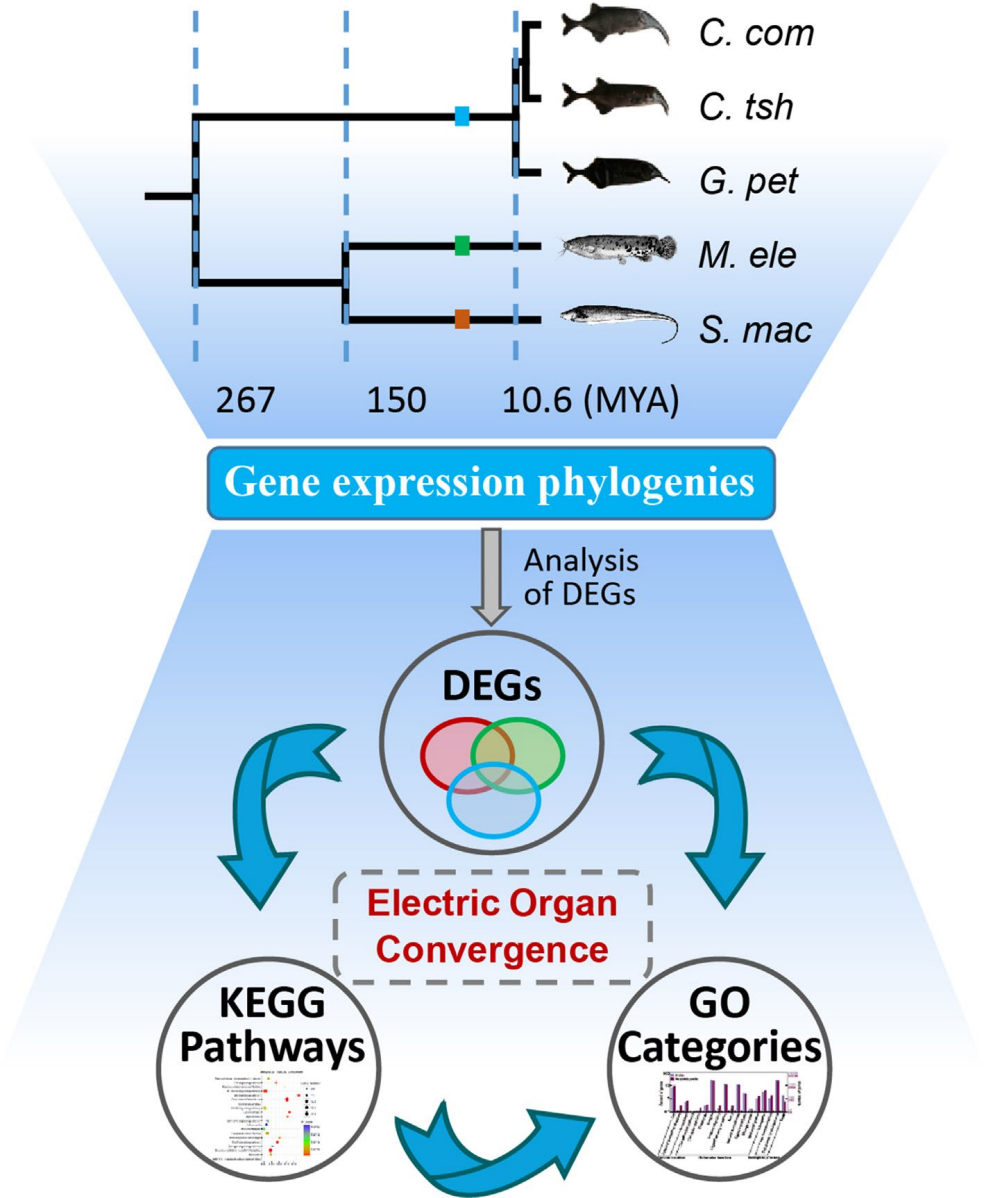
Analysis of genetic mechanism underlying phenotypic convergence of electric organs among electric fishes. The species tree was obtained from previous studies (Lavoue et al., 2012; Gallant et al., 2014; Lamanna et al., 2015) and the TimeTree database (Hedges et al., 2015). Fish images obtained from Lamanna et al. (2015) or FishBase (https://www.fishbase.se/home.htm). EO and SM represent the electric organ and skeletal muscle, respectively. *C. com*, *C. tsh*, *G. pet*, *M. ele*, and *S. mac* represent *C. compressirostris*, *C. tshokwe*, *G. petersii*, *M. electricus*, and *S. macrurus*, respectively. We used these same abbreviations for all the following figures and tables.

All Illumina reads used for this study were downloaded from the Sequence Read Archive (SRA, http://www.ncbi.nlm.nih.gov/sra) (see **Table S1** for detailed information) (Gallant et al., 2014; Lamanna et al., 2015). We focused on five species from three lineages (Mormyroidea, Siluriformes, and Gymnotiformes), including *Campylomormyrus compressirostris*, *Campylomormyrus tshokwe* and *Gnathonemus petersii*, *Malapterurus electricus*, and *Sternopygus macrurus*, respectively. Quality control of raw reads was performed with FastQC (v0.11.5). After clipping Illumina adapter sequences and trimming low-quality bases using fastx-toolkit, the clean reads were *de novo* assembled using Trinity (v2.2.0) (Grabherr et al., 2011; Haas et al., 2013) with default parameters to construct unigenes. Short reads were individually mapped to their respective transcriptome assemblies using Bowtie2 (v2.2.8) with default parameters. To estimate gene expression levels, clean reads from the EO and SM were mapped to non-redundant unigenes to calculate the fragments per kilobase transcript length per million fragments mapped (FPKM) value using RNA-Seq by Expectation-Maximization (RSEM) (v1.2.29) (Li and Dewey, 2011). These expression values were normalized for sequencing depth and transcript length and then scaled *via* the trimmed mean of M values (TMM) normalization under the assumption that most transcripts are not differentially expressed. The further analysis process of our study is shown in **Figure 1**.

### Orthologous Gene Assignment

To reduce the effects of potential isoform variation among species, here we kept only the longest unigene for further analysis. The putative orthologous relationships of genes were determined according to the following process. First, we used OrfPredictor to predict protein-coding sequences of unigenes (Min et al., 2005), and only the unigenes predicted as protein-coding genes were kept for further analysis. Second, we clustered the gene predictions of the five focal species into orthologous gene groups (OGGs) using OrthoMCL (Li et al., 2003). Third, for each OGG, we selected the most similar pair among the five species, which resulted in 7,716 OGGs with high confidence for facilitating future comparative analysis.

### Hierarchical Clustering Analysis and Principal Component Analysis

Based on the orthologous relationship, we obtained a final matrix consisting of expression values for 7,716 OGGs in 16 samples. Moreover, the FPKM matrix was log2-normalized after adding a pseudocount of 0.01. Samples were clustered hierarchically based on their pairwise Spearman’s correlation coefficients (ρ) of gene expression values using the complete linkage clustering algorithm applied on Euclidean distances (Breschi et al., 2016). Hierarchical clustering analysis and principal component analysis (PCA) were conducted using R.

### Gene Expression Phylogenies

The gene expression data set was used for building a distance matrix based on pairwise distance matrices (1-ρ) (Brawand et al., 2011; Ruan et al., 2016) and then subjected to neighbor-joining tree constructed using functions in the ape package (Paradis et al., 2004) in R (v3.5.2). The reliability of branching patterns was assessed with bootstrap analyses (randomly sampled with replacement 1,000 times) using TreeExp 1.0 package in R (Ruan et al., 2016). The bootstrap values indicate the proportions of replicate trees that share the branching pattern of the majority-rule consensus tree. Other distance matrices, including Euclidean distance and angular cosine distance, were also used for gene expression phylogenies (Ruan et al., 2016).

### K-Means Co-Expression Clustering

To determine the similarity of gene expression among different samples, we used the K-means clustering algorithm in R to cluster gene expression data. First, the gene expression value FPKM was transposed to log2. To avoid the situation where FPKM equals zero, we added 0.01 to all the expression values and merged the transposed expression values into a matrix. We clustered the expression matrix into 8∼32 gene clusters and calculated Spearman’s correlation coefficient among the EOs, SMs, and EOs and SMs in each gene cluster with the R built-in function. The significance of the difference was determined using Student’s *t*-test.

### Analysis of DEGs

DEG between the EO and SM was analyzed using the Bioconductor package edgeR (Empirical analysis of Digital Gene Expression in R) (Robinson et al., 2010). The calculated *p* values were subjected to the Benjamini–Hochberg method (Benjamini and Hochberg, 1995) to control for false discovery rate (FDR). In this study, FDR ≤ 0.05 and fold change (FC) ≥ 2 were set as the thresholds to determine the significance of DEGs.

### Comparative Analysis of Functional Enrichment for DEGs

KO-Based Annotation System (KOBAS) 3.0 (http://kobas.cbi.pku.edu.cn/) is a web server for functional annotation and functional set enrichment of genes (Xie et al., 2011). We used BLASTx in BLAST + v2.3.0 (Camacho et al., 2009) for functional annotation of transcriptomic sequences for each species based on best hits (*E* value ≤ 10^-5^) to zebra fish sequences deposited in web Ensembl, and implemented GO (gene ontology) and Kyoto Encyclopedia of Genes and Genomes (KEGG) pathway enrichment analysis on KOBAS 3.0. Statistical significance was calculated using a hypergeometric test with an FDR correction method (Benjamini and Hochberg, 1995), and the threshold was set as FDR less than 0.05. To determine whether there is a higher similarity among DEGs at the level of functional composition, comparative analyses of GO term and KEGG pathway enrichment analyses were conducted for DEGs between the EO and SM tissues among five species. We performed Fisher’s exact tests between pairs of species to determine whether there was a statistically significant number of common GO terms and KEGG pathways. Here, we set the shared DEGs from all DEGs between any two species or among all species as background. Compared with the corresponding background, we employed Fisher’s exact tests between pairs or all across species to determine whether there were statistically significant numbers of shared GO terms or KEGG pathways.

## Results

### Lineage Rather Than Organ Clustered Together by Hierarchical Clustering and PCA

We downloaded 16 libraries (8 each from EOs and SMs) from previous reports (Gallant et al., 2014; Lamanna et al., 2015). After quality filtering, we constructed *de novo* transcriptome assembly for each species separately using Trinity (Grabherr et al., 2011; Haas et al., 2013). The detailed gene information is listed in **Figure S1**. Although *de novo* assembly enables functional genomic studies, it has the potential for mis-assemblies and consequent biases in downstream analyses. To exclude probable exogenous RNAs and thus provide evidence that these genes are “true” genes but not sequencing or assembly artifacts, only genes showing a significant BLAST hit for at least one of the 12 published fish genomes (see “Materials and Methods”) were kept for further analysis (**Figure S1A**). Owing to the small sample size, the results of *de novo* assembly were not perfect, but it was still fit for analysis in the present study.

**Figure 2:**
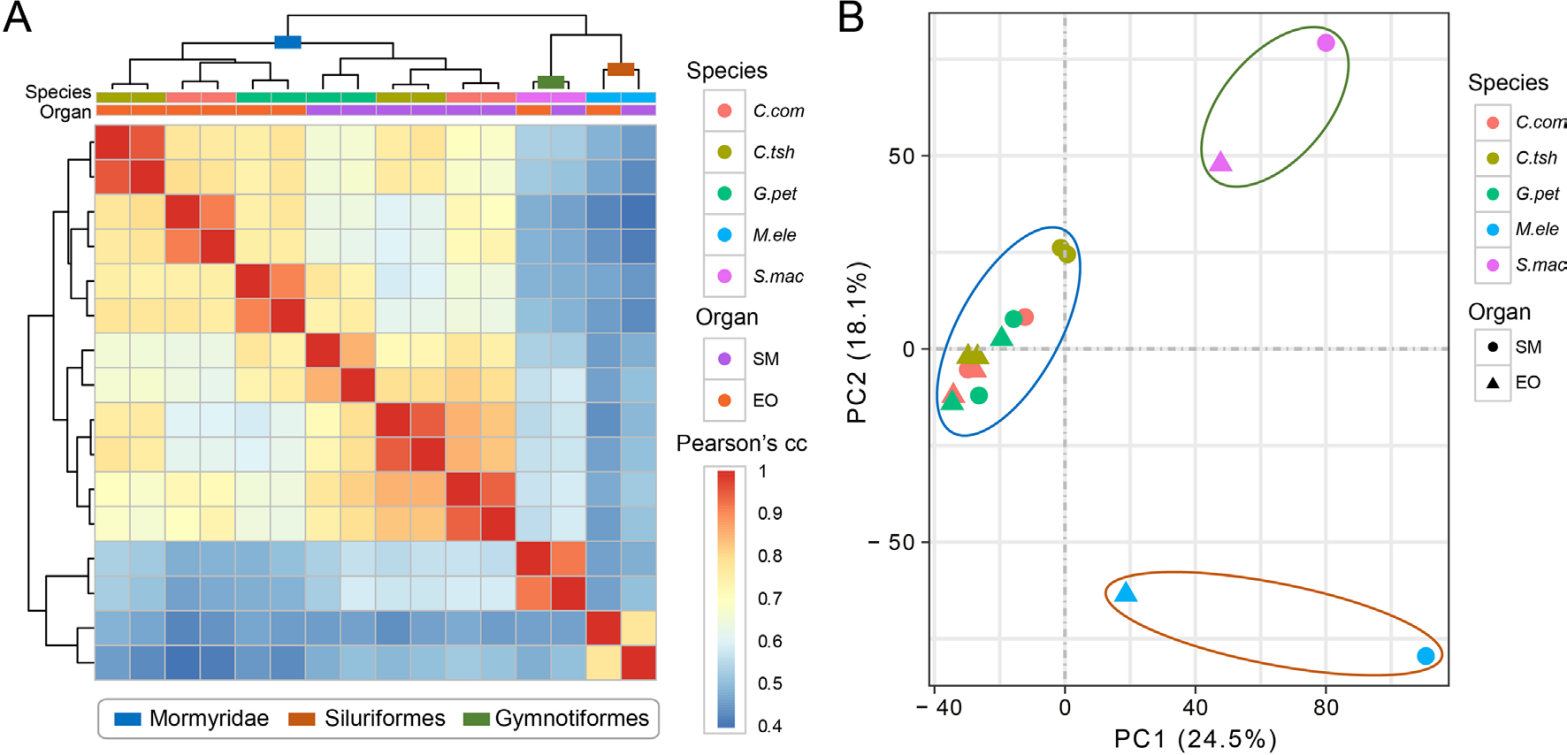
Hierarchical clustering and PCA analysis of electric fish tissues. **(A)** Clustering and heat map analysis were performed using R software to cluster samples into hierarchies using pairwise Spearman’s correlation coefficient applied on Euclidean distances. The correlation coefficients are represented as a heat map, indicating variation ranging from high (red) to low (blue) similarity. **(B)** The PCA for all the 16 tissue samples included this study was based on the expression values of transcriptome-wide expression profiles, which determine the key variables within the data set that explain the differences between the samples.

We used gene expression values that were estimated by RNA-seq of the EO and SM tissues in five different electric fishes from three independent lineages to determine the expression differences among tissues and species. Here, we restricted the analyses to the set of 7,716 OGGs that could be identified as orthologs across the five species and used log-transformed expression values. To obtain an initial overview of gene expression patterns, we performed hierarchical clustering for the expression matrix of 7,716 OGGs. We found that the samples cluster preferentially by lineage (Mormyroidea, Siluriformes and Gymnotiformes). As shown in **Figure 2A**, two organs from each lineage group together, and the three species from the Mormyridae lineage clearly separate the data according to tissues. The former indicates that the expression of the EO shows totally distinct patterns from independent origins. Moreover, the latter may reflect the ancient divergence of these two tissues dating to the Mormyridae common ancestor, because closely related species have more similar expression levels and gene expression patterns of homologous traits from common evolutionary origin are evolutionarily conserved. Additionally, our result of PCA also provided evidence for the pattern where two organs from each lineage clustered together (**Figure 2B**).

### The Expression Phylogeny of Transcriptome-Wide and Function-Related Genes Evolved by Lineage Independently

First, we investigated the divergent time among species mainly based on the TimeTree database (Hedges et al., 2015) and Lavoue’s report (Lavoue et al., 2012). The divergence of *Sternopygus* and *Malapterurus* occurred about 150 million years ago (Mya) (95% credibility interval [CI] = 116–176 Mya) (Hedges et al., 2015). The Mormyroidea and Gymnotiformes, the African and South American weakly electric fishes, respectively, occurred well after the splitting of the respective lineages from their most recent common ancestor about 267 Mya (CI = 236–298 Mya). The divergent time of the *Campylomormyrus* and *Gnathonemus* was estimated to be 10.6 Mya (Lavoue et al., 2012; Hedges et al., 2015). Hence, we obtain an illustration of the species tree as shown in **Figure 1** according to the above information.

**Figure 3:**
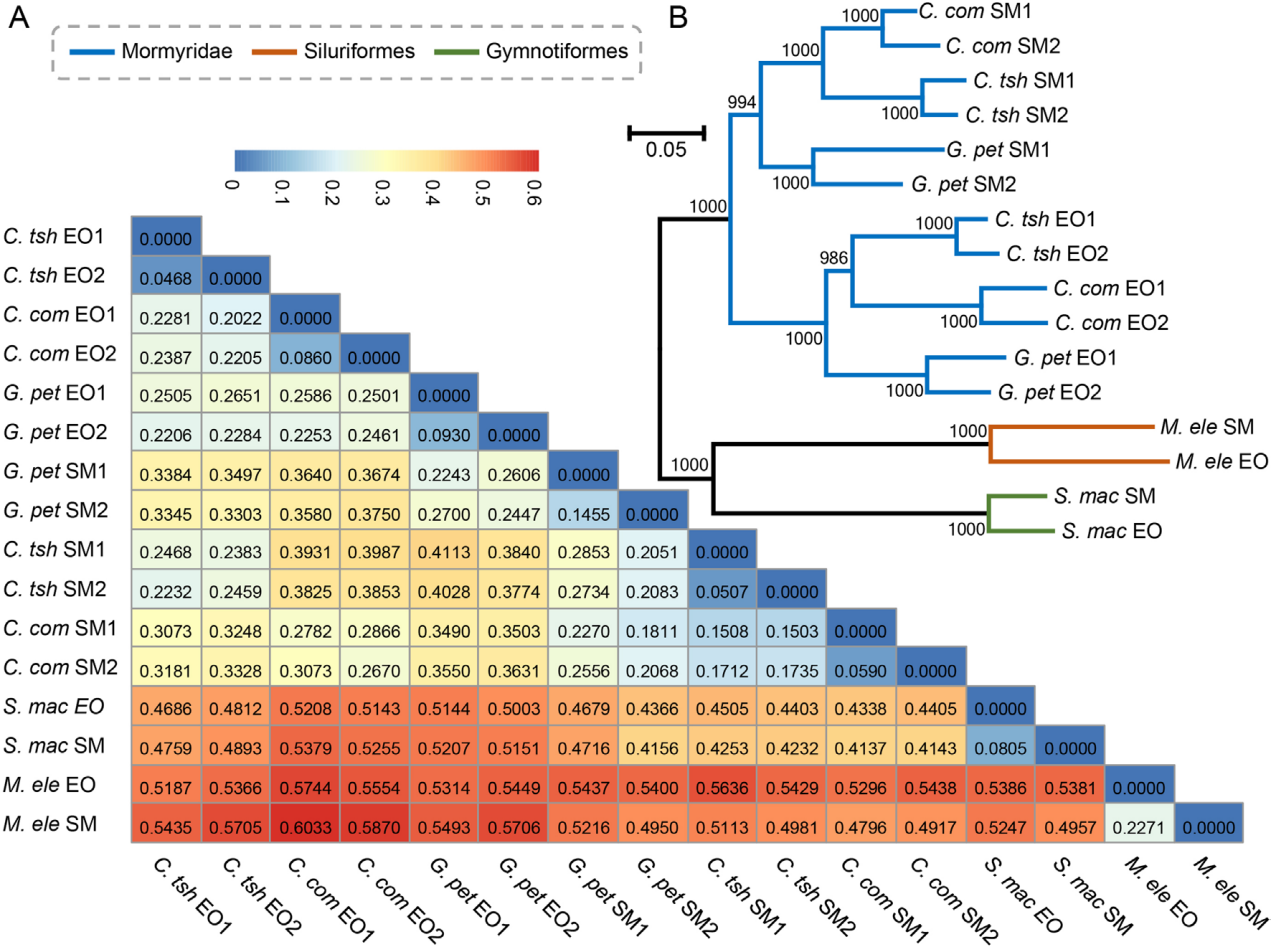
Paired distance matrix and expression phylogenetic analysis of EO and SM expression profiles. **(A)** Based on the gene expression profiles of the SMs and EOs, a paired distance matrix was constructed based on pairwise distance matrices (1-ρ). The ρ here means Spearman’s correlation coefficient. **(B)** NJ analysis of expression level of the SM and EO based on the matrix in part A. The numbers following EO or SM denote the sample replicate.

Next, we obtained pairwise distance matrices (1-ρ) based on expression levels in the EO and SM tissues (**Figure 3A**). Neighbor joining (NJ) expression phylogeny of the SM and EO was reconstructed based on this distance matrix. It exhibited a similar result to those of hierarchical clustering and PCA, which separate the data according to evolutionary lineages with high bootstrap values (**Figure 3B**). The expression phylogenetic relationships generated by the expression matrix agree with the known species phylogeny, and also with recent reports on phylogeny for these three lineages (Lavoue et al., 2012; Gallant et al., 2014; Lamanna et al., 2015). **Figure 3B** strongly shows three independent origins of the EO among three lineages, with the EO branch being a sister to the SM branch. Similar to the result of hierarchical clustering, the Mormyroidea lineage was split into two natural groups, one consisting of all the EO tissues and the other of all the SM tissues (**Figure 3B**), suggesting that the gene expression has changed during the evolutionary process from the SM to the muscle-derived EOs. A similar topology was also presented as phylogenies based on Pearson distance, Jaccard distance, Euclidean distance, and angular cosine distance, respectively (**Figure S2**). Thus, evolutionary signals inherent in our data, which may reflect changes in cellular gene expression levels or changes in the cellular composition of organs between species, outweigh the gene expression variability arising from sampling differences.

These observations prompted us to determine whether these genes related to tissue-specific function were responsible for the convergent traits. After GO annotations conducted for these 7,716 OGGs, in this step, we only selected the genes following GO terms related to muscle contraction, ion binding, motor activity, axon guidance, and embryonic development for further analysis. These genes are potentially involved in the development and/or tissue-specific function of either SM or EO (Gallant et al., 2014; Lamanna et al., 2014). Thereby, we identified 696 candidate genes with complete or partial CDS (coding sequence). However, after the phylogeny analysis, almost the same topology (**Figure S3A**) was obtained with the expression matrix of 7,716 OGGs. From this, we may infer that the repeated emergence of the EO from different lineages is not the result of the same or similar ways of expression alteration.

Finally, we built an expression distance matrix for each tissue and reconstructed gene expression trees, respectively (**Figures S3B, C**). The topologies of both the EO and SM gene expression phylogenies were roughly congruent with the electric fish phylogeny, and they correctly resolve the three major electric fish lineages (Mormyroidea, Siluriformes, and Gymnotiformes) (**Figure 1**). Those small differences may result from bias such as the influence of age, feeding status, and other characteristics of the individuals, which could not be perfectly matched between species, for practical and biological reasons. Overall, these results may bolster the conclusion that there was no transcriptome-wide convergence presenting in EOs from the three distinct lineages.

### There Is No Convergence of Large-Scale Gene Expression

Our analysis of the expression profiles of EO and SM tissues showed that there was no convergence in the expression of EO between different lineages, indicating that the EOs of different lineages had completely different expression types. On the whole, the degree of differentiation of the organizations in the lineages did not exceed the degree of differentiation between the lineages. Then, is it possible for some of these genes to show convergence? We clustered the obtained one-to-one expression profiles by K-means and found no obvious similar expression patterns in the EOs among the 12 gene clusters (**Figure S4**). We also used the K-means clustering to divide the expression spectrum into 8∼32 gene clusters, and the result was the same. To a certain extent, this reflects that there are not quite a number of gene clusters with similar expression types in the EOs from different origins. In other words, most homologous genes present different expression types in the lineage of electric fish originated independently.

To further evaluate the correlation of gene expression types in each gene cluster, we tested the correlation of different expression levels among 12 gene clusters. We calculated the correlation coefficients of expression profiles between the EOs (**Figure S5**, EO), the correlation coefficients between the SMs (**Figure S5**, SM), and the correlation coefficients (**Figure S5**, E/S) between the EO and SM in each gene cluster. Then, Student’s *t*-tests for any two of these three sets of data were performed, and the significance is shown in the figure. Although the correlation between the expressions of the EOs is significantly higher than that between the other two sets, not all the EO samples have a higher correlation than other samples, and thus, it is still unable to explain the convergence of gene expression in the EOs. Therefore, we conclude that there is no large-scale convergence of gene expression in EOs of different lineages.

### Fewer Shared DEGs Identified Among All Five Electric Fishes

**Figure 4:**
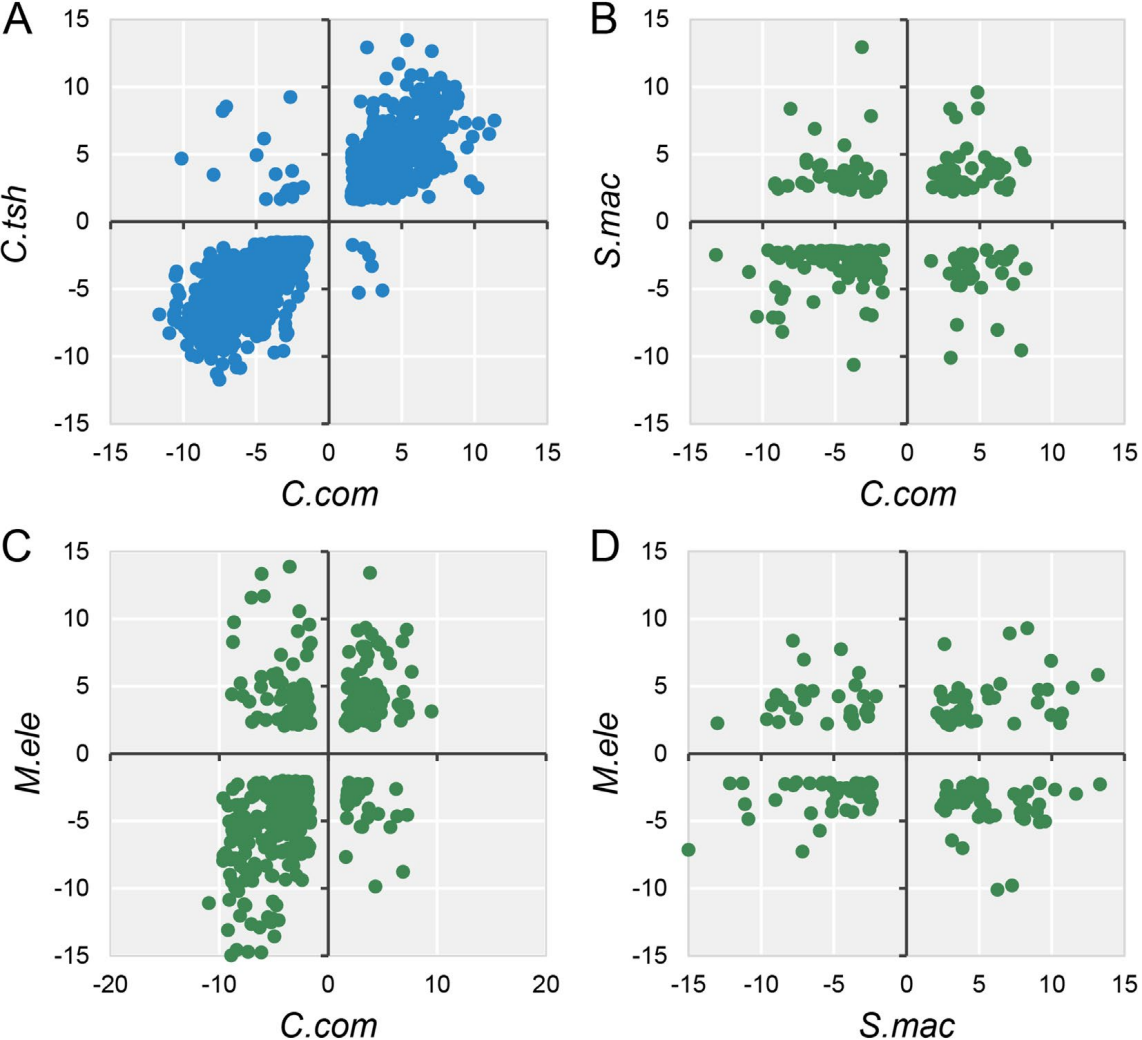
Log2 fold expression change correlation between different species. The dots mean the expression fold change between EO and SM of DEGs (FDR ≤ 0.05 and FC ≥ 2) in any two species, and quadrants 1 and 3 of the scatter graph exhibit the conserved directionality of differential expression (both species in the EO exhibit higher expression or both species in SM exhibit higher expression) compared to one another. Blue **(A)** and green **(B**, **C**, and **D)** dots indicate intra-lineage and inter-lineage species comparisons, respectively. Here we only selected four representative comparisons to be shown, and the others are shown in **Figure S7**.

Accordingly, during the specialization of myogenic tissues to EO, the electrocyte morphology has changed greatly with myogenic cells, including large cell size, increased excitability, decreased contractility, and enhanced insulation (Gallant et al., 2014). Given that phenotype difference, we performed differential expression analysis for EO and SM tissues in each species, with the criteria (FDR ≤ 0.05 and FC ≥ 2). All species but *G. petersii* have comparable DEG numbers (**Figure S6A**). Compared to the DEG number of each species, the shared DEGs between any two species from different lineages only account for a small proportion (**Figure S6B**). To investigate whether these EO convergence–related species exhibit parallel patterns of gene expression differentiation (possibly associated with a similar living environment), we compared DEGs between EO and SM from all five species, using only one-to-one OGGs that satisfied our minimum differential expression criteria in all samples. The fact is that except for *M. electricus* and *S. macrurus*, no less than half of shared DEGs exhibited the conserved directionality of differential expression (both species in the EO exhibit higher expression or both species in SM exhibit higher expression) compared to one another (**Figures 4** and **S7**), supporting the proposition that parallel environment differentiation reflects parallel adaptation. Moreover, pairwise species comparisons revealed that gene expression divergence increases overall with evolutionary time (**Figure S6**).

**Figure 5:**
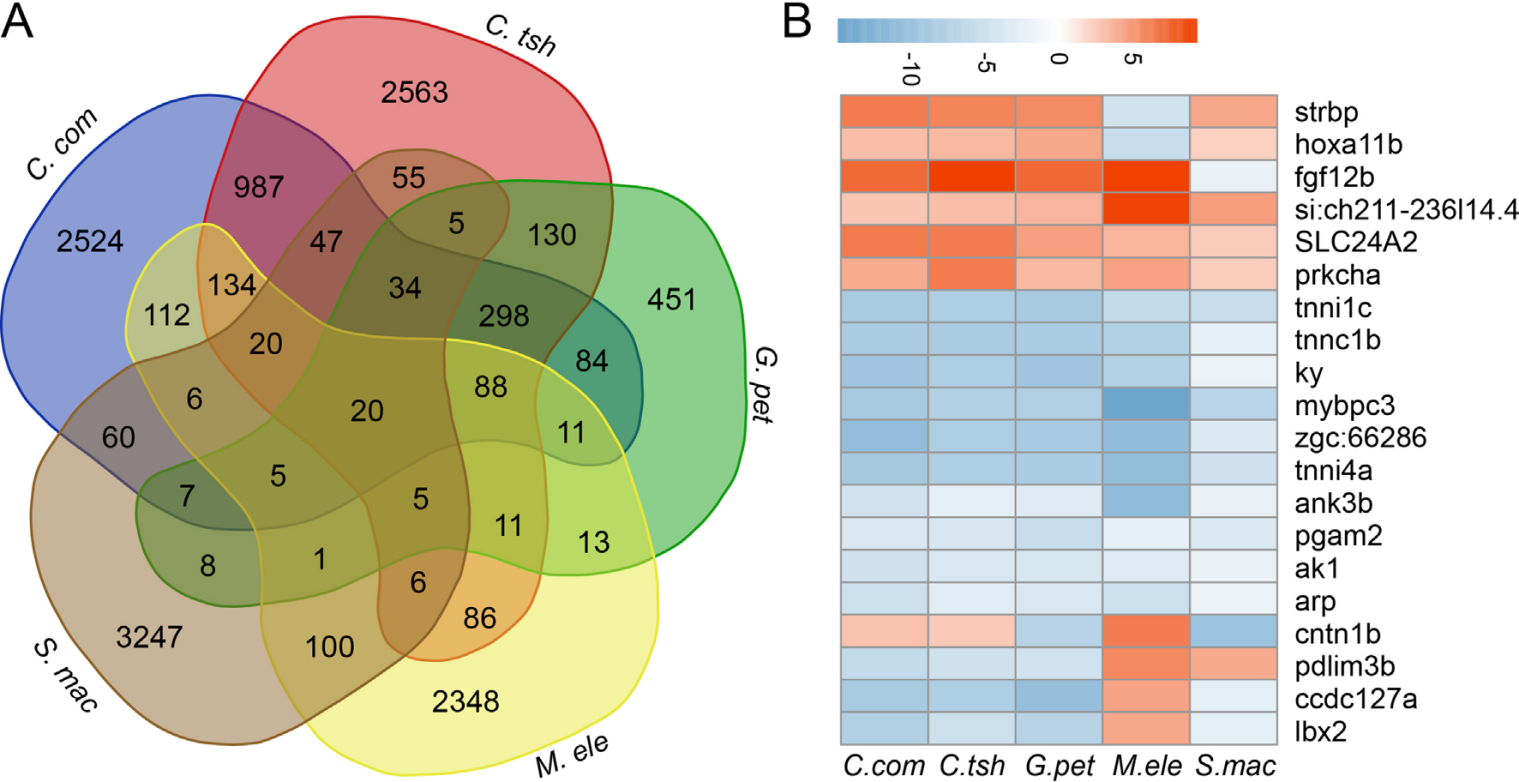
Comparison analysis of DEGs between SM and EO tissues among five electric fishes. **(A)** Venn diagram of DEG (differentially expressed between EO and SM) among five electric fishes. The number in the figure represents the DEG number. Venn diagram analysis was conducted by online tool (http://bioinformatics.psb.ugent.be/webtools/Venn/). **(B)** The illustration shows the log2 fold change of 20 shared DEGs across the five focal species (red, upregulated in EOs, and blue, downregulated in EOs).

Furthermore, although large numbers of genes showed differential expression, there were quite few shared DEG sets (**Figures 5A** and **S6**). In fact, when all five species were considered, there were only 20 genes, including *strbp*, *pdlim3b*, *tnni1c*, *lbx2*, and other genes, showing significantly different gene expression between the EO and SM tissues (**Figure 5B**). Despite the relatively small number of shared DEGs, their directionality (upregulation in EO or SM samples) was not well conserved across species, among which only 13 genes exhibited the same direction of differential expression (**Figure 5B**). These 13 genes play important roles in ATP binding, ion transport, and SM contraction. Hence, we speculate that the expression change of these genes may affect tissue morphology, which then facilitates the generation of novelties.

### Different Genes but Shared Functions Involved in Electric Organ Convergence

**Figure 6:**
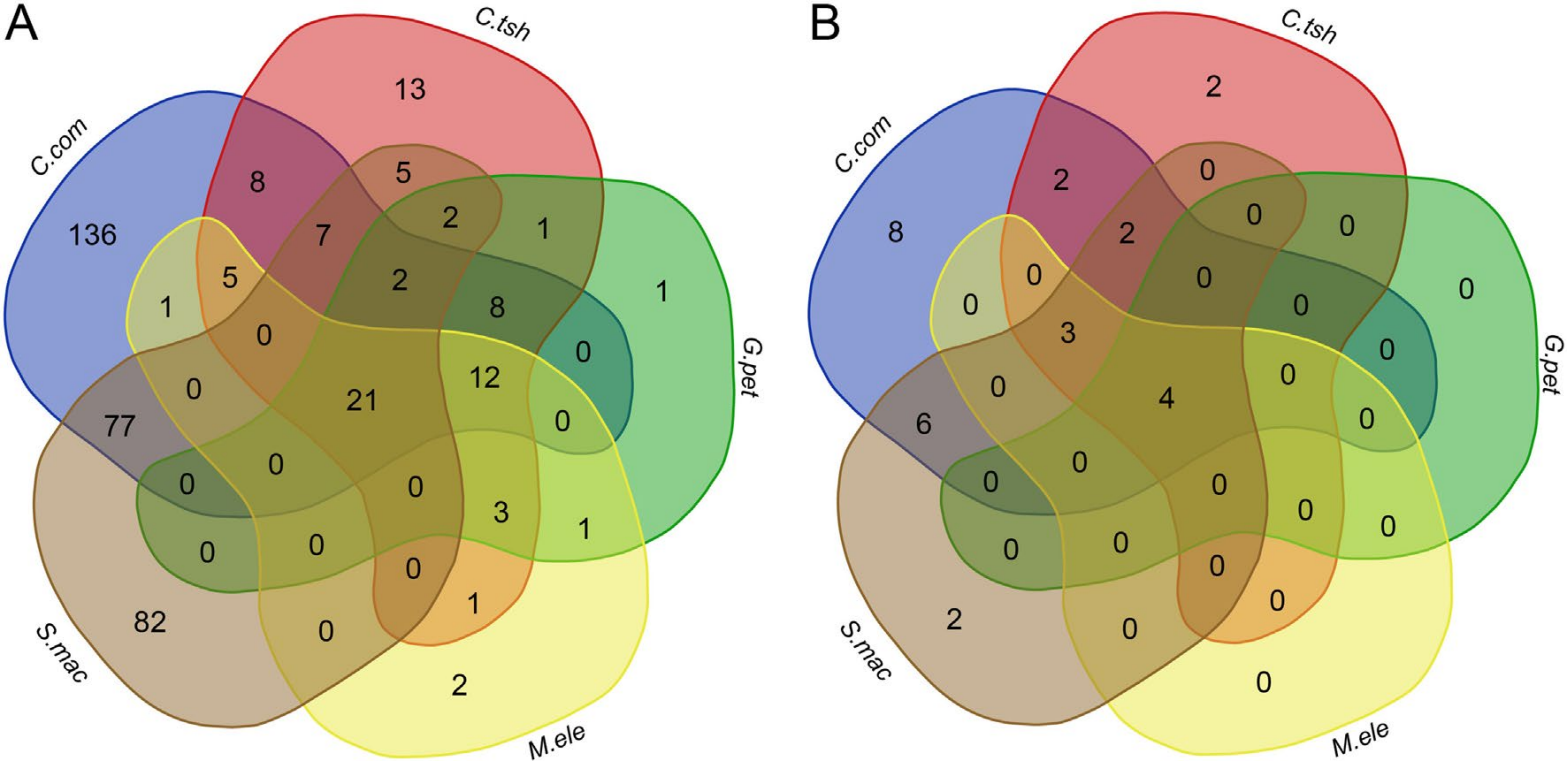
Comparison analysis of GO categories and KEGG pathways for DEGs between the SM and EO tissues among five electric fishes. **(A)** Venn diagram of the significantly enriched GO categories for DEGs among five electric fishes (FDR < 0.05). The numbers represent the number of significantly enriched GO categories. **(B)** Venn diagram of the significantly enriched KEGG pathways for DEGs among five electric fishes (FDR < 0.05). The numbers represent the number of significantly enriched KEGG pathways.

To determine whether there is a higher similarity of DEGs at the level of functional composition, we compared GO term and KEGG pathway enrichment analyses for the DEGs between the EO and SM tissues among five species. Intriguingly, we found that there were 21 GO categories of consistently enriched DEGs (FDR < 0.05) across five electric fishes (**Figure 6A**, **Table S2**), including muscle system process, sarcomere, multicellular organismal process, single-multicellular organism process, striated muscle thin filament, actin cytoskeleton, myofibril, calcium ion binding, myofilament, single-organism developmental process, striated muscle contraction, muscle contraction, developmental process, system development, SM contraction, anatomical structure development, multicellular organism development, troponin complex, actin binding, cytoskeletal protein binding, and tissue development. This may indicate that these similar function modules play pivotal roles across species. Fisher’s exact test showed a significantly higher overlap level in the number of GO categories than DEGs among all focal species (**Table S3**, *p* value < 0.05). We also performed Fisher’s exact tests for pairwise comparison, which suggested that all pairs but *C. compressirostris* and *C. tshokwe* showed a statistically significant overrepresentation.

At the KEGG pathway level, there were four shared pathways across five species (FDR < 0.05, **Figure 6B**, **Table S2**), including adrenergic signaling in cardiomyocytes, tight junction, calcium signaling pathway, and cardiac muscle contraction, respectively, which imply that expression alternation in many different genes can change the phenotype in similar ways. Fisher’s exact test showed significantly more KEGG pathways shared by five focal species (**Table S3**, *p* value < 0.05). Of particular interest is that the Fisher’s exact tests performed for pairwise comparison suggested that all pairs from different lineages significantly presented more shared pathways. But for pairwise comparison among *C. compressirostris*, *C. tshokwe*, and *G. petersii* (**Table S3**, *p* value > 0.05), it was less significant because they were derivations from a common evolutionary origin. Thereby, all these shared genetic networks and pathways may contribute to the morphology changes during this specialized process from myogenic tissues to the EOs, including cell size, excitability, contractility, and insulation (Gallant et al., 2014). Taken together, to a certain extent, these may raise the issue that different genes performed the same functions and pathways to achieve the phenotypic convergence. Hence, we speculated that different genes but similar functional modules or pathways from distinct lineages contribute to the tissue morphology changes and then facilitate the generation of novelties.

## Discussion

Generally, changes in gene expression are thought to underlie many of the phenotypic differences between species (Brawand et al., 2011). Gene expression pattern may have altered to adapt to function change following environmental challenges and selection pressures. Previous studies proposed that owing to the contingency in natural environment, in several cases, similarity of evolutionarily convergent traits may represent the same or similar evolutionary paths to achieve their goals, such as complex bioluminescent organs of squid (Pankey et al., 2014). In other words, if natural selection acted on an optimal phenotype, widespread changes in gene expression could readily generate similar phenotypic traits over a certain evolutionary time regardless of unrelated origins (Whittall et al., 2006; Reed et al., 2011; Jones et al., 2012). A homologous precursor trait evolved in parallel among different lineages leads to massively parallel evolution of gene expression accompanying separate origins of traits (Pankey et al., 2014). However, in our study, we failed to detect transcriptome-wide convergence presenting in the EOs across different electric fish lineages, which means that fewer genes maintain a similar expression pattern from three distinct lineages. One may propose that the species can possess very similar gene expression patterns important for electrogenesis at their ancestor states, but only partial expression patterns most essential for the novel trait were survived after a long-time divergence. These signals might be overwhelmed by the random background noise during the evolutionary process. However, our phylogenetic analysis suggested that the evolutionary process of the EO derived from the muscle was likely to have taken place after divergence of three lineages (**Figure 3B**), and even the function-related genes showed distinct expression patterns (**Figure S3A**). Therefore, it was less likely to determine the next evolutionary direction after divergence from the common ancestor. Therefore, the emergence of EO from different lineages may result from different genetic alterations.

Actually, unless exposed to strong constraints, complex organs are less likely to originate multiple times independently following the same or similar trajectories (Abouheif and Wray, 2002; Kronforst et al., 2012; Pankey et al., 2014). As a consequence, this may not reflect a general statement about the mechanisms of convergent traits. Gene expression patterns of homologous traits are evolutionarily conserved under the intricate functional requirements (Chan et al., 2009; Brawand et al., 2011). In contrast, they should be less correlated for convergent traits owing to their different evolutionary origins (Tanaka et al., 2009; Emera et al., 2012). Hence, despite the phenomena of convergence being frequently observed, it is difficult to detect the rules underlying the convergent outcomes. In our study, we found that the gene expressions of two organs from distinct lineages tend to group together within lineage, rather than organ (**Figures 2** and **3B**), which may indicate that there was no parallel evolution of the entire transcriptome among the EOs from different lineages. Although more than half of DEGs showed the same direction of differential expression both within lineages and between lineages (**Figure 4**), the relatively few shared DEGs were identified across all focal species, which may bolster the issue that parallelism for expression phenotypes results from functionally varying selection (Zhao et al., 2015). However, GO and KEGG pathway enrichment analyses confirmed that there were more overlaps of DEGs at the level of biological function (**Figure 6** and **Table S3**). Our study illustrates that this novel phenotype from divergent lineages might arise from the combination of expression alternation of multiple genes from the same genetic pathways, rather than from a similar change of single gene expression with large effect. Therefore, the outcome of our study supported the latter explanation, according to which the phenotypic convergent evolution of certain traits was characterized through multiple solutions for the mechanisms underlying the EO convergence.

Therefore, the phenotypic convergent evolution of certain traits shaped through multiple different genetic mechanisms in divergent taxa tends to be more widespread. Several lines of evidence from previous reports also support this hypothesis at the level of both gene or protein sequence and gene expression. For instance, in eusocial insects, Berens et al. only identified a dozen common caste DETs (differentially expressed transcripts) but more shared gene networks related to eusocial evolution among the three hymenopteran social lineages (Berens et al., 2015). This demonstrated that different expression patterns but conserved pathways contributed to the convergence of caste phenotypes among lineages (Berens et al., 2015). Therefore, although different genes were involved in this process, the similar ultimate goal can be obtained based on the similar pathways and biological functions. Strikingly, this can also be observed at the protein sequence level. For instance, Mirceta et al. have elucidated this phenomenon focusing on myoglobin in mammalian diving capacity (Mirceta et al., 2013). They found that the net surface charge of myoglobin (*Z*_Mb_) in mammals is positively related to the mammalian diving capacity, which means that convergent evolution can take place through the change of two distinct amino acid clusters (Mirceta et al., 2013). Hence, any change of amino acid leading to the increase of *Z*_Mb_ can be regard as an adaptive mutation, which can provide a reasonable explanation of why all the marine mammals evolved independently but with higher diving capacity.

It is noteworthy that the original data used were published independently and not designed to be directly compared. Although there must be a number of differences between these studies and there was a low sample size, we were still able to uncover significant overlaps from our analysis. In our study, we conducted our analysis using the EO and SM transcriptome of five species from three lineages, with three species from one lineage and the other two from two independent lineages. Although Galant et al. reported *S. macrurus* to be least similar to other gymnotiforms (its EO transcriptome shows little to no downregulation of myogenic genes, whereas EO transcriptomes of Eigenmania and Electrophorus studies showed significant downregulation of SM genes in their EOs) (Gallant et al., 2014), it is independently originated from lineages of Siluriformes and Mormyroidea. The use of *S. macrurus* may introduce a bias, but theoretically, it cannot affect the main conclusion of our study. Because there was no reference genome for all the electric fishes but eel, adding more species would reduce the number of OGGs among all investigated species. To avoid this problem, we added three species of Mormyroidea from Lamanna et al. (2015) to compare differences between species in a lineage and selected one species from Gymnotiformes to compare differences between lineages. From our analyses, although many shared DEGs were observed between any two focal species, only twenty overlapping genes were present in all species (**Figure 5**). However, our results implied that similar functional changes are, to a certain extent, associated with the convergent trait of generating electricity within shared functional modules and pathways. This may indicate that different lineages confer divergent genetic responses to environmental challenges, and which mechanism was chosen largely depended on the precise selection pressure (Stern, 2013). Accordingly, varied genes can perform similar functions, and selection likely constrained function within a limited set of gene networks and pathways (Berens et al., 2015). During this evolutionary process, the same genes are not necessarily involved in similar EO convergence across different origins of electric fishes. Therefore, among the convergent traits, which genes were recruited to change their expression and the extent of the expression changes in parallel may largely rely on the trade-off between the developmental constraints and the selection pressure in directing phenotypic evolution (Losos, 2011).

## Conclusion

To understand the dynamics of electric fish transcriptome evolution, in our study, we reanalyzed the transcriptomic data from the EOs and SM across five species and found that the gene expression of these two organs from three lineages preferentially group together within lineage, rather than within organ. In the comparison of the expression of DEGs with one another, the results indicated that no less than half of genes showed parallel expression differentiation with the same direction of differential expression irrespective of their lineages. Although fewer shared DEGs among all focal species were identified, these species contained more overlaps at the level of biological function and pathways. In light of this, we conclude that there was no parallel evolution of the entire transcriptomes among the EOs from different lineages and different genes, but it was conserved molecular functions that led to the convergence of this complex trait. Actually, unless subjected to strong constraints, the probability of complex traits originating independently through similar trajectories should be very small. However, it can be much easier for divergent lineages to provide convergence of organismal adaptation and then increasing this fitness through multiple solutions, as in the saying “All roads lead to Rome.” Therefore, this led us to support the hypothesis that the phenotypic convergent evolution of certain traits shaped through different evolutionary paths tends to be a general rule underlying the phenotype convergence and may imply that the power of natural selection can shape the convergent adaptations from unrelated starting points.

## Author Contributions

AL, ZS, and XG designed the study. AL and FH carried out the experiments and analyses with the help of JZ, YZ, and ZS. AL, FH, and JZ drafted the manuscript. All others contributed to the revision of the manuscript. All the authors read and approved the final version of the manuscript.

## Funding

This study was supported by a grant from the National Natural Science Foundation of China (31571355) and the Fund for Shanxi “1331 Project” Key Subjects Construction (1331KSC).

## Conflict of Interest Statement

Author ZS was employed by company Singlera Genomics Inc. The remaining authors declare that the research was conducted in the absence of any commercial or financial relationships that could be construed as a potential conflict of interest.

## Supplementary Material

Figure S1 The transcriptome assembly statistics assembled de novo with Trinity.

Figure S2 Phylogeny of gene expression based on different vector distances. A-D were constructed based on Pearson distance, Jaccard distance, Euclidean distance, and angular cosine distance, respectively.

Figure S3 Expression phylogeny of functionally related genes and the EOs and SMs. A. The NJ analysis based on 696 functionally associated genes. The NJ trees of gene expression phylogenies for the SM (B) and the EO (C). The tree topologies were calculated based on pairwise distance matrices (1-ρ).

Figure S4 Co-expression and clustering of the EO and SM in electric fish. The gene number of each gene cluster is shown in the lower left corner. The white polyline represents the median centralized expression value of a single gene log2 in each sample, and the red polyline represents the median value of the gene cluster in different samples. The background colors of blue, green, and red represent three species of Mormyroidea, Siluriformes, and Gymnotiformes, respectively. The left part is the SM, and the right part is the EO.

Figure S5 Comparative analysis of Spearman’s correlation coefficient between 12 expression clusters in different tissues. EO, SM, and E/S represent the correlation coefficients between any two expression profiles of the EOs, SMs, and between EO and SM, respectively. * denotes p < 0.05; ** p < 0.01; *** p < 0.001.

Figure S6 Comparative analysis of DEGs among five electric fishes.

Figure S7 Log2 fold expression change correlation between different species.

Table S1 The detailed information of raw data used for this study.

Table S2 The GO and KEGG pathway enrichment analyses for DEGs between the SM and EO in each electric fish.

Table S3 Fisher’s exact test for statistically significant overrepresentations of GO categories and KEGG pathways of shared DEGs among different lineages.
