# Supplementary material for: Comparative Transcriptome Analyses Reveal the Role of Conserved Function in Electric Organ Convergence Across Electric Fishes
Source: Front Genet. 2019 Jul 18;10:664. doi: 10.3389/fgene.2019.00664 (PMC6657706; doi:10.3389/fgene.2019.00664)
Supplement: Figure S1 — The transcriptome assembly statistics assembled de novo with Trinity. [file Image_1.pdf]

	S. mac	*M. ele*	*G. pet*	*C. tsh*	*C. com*
**Total number of transcripts**	305,467	144,326	208,642	254,753	193,201
**Total number of genes**	256,364	128,751	180,377	218,251	167,006
**Total assembled bases**	178,411,183	90,922,897	110,061,126	135,303,701	102,226,573
**Median length of genes**	392	398	347	352	347
**Mean length of genes**	695.93	706.19	610.17	619.95	612.11
**N50 value of genes**	1017	1076	861	869	866
**Percent GC (%)**	46.20	43.54	48.12	47.86	48.39
**Predicted Genes**	53,068	45,483	44,312	44,073	42,077
