# Supplementary figures and images for: Comparative Transcriptome Analyses Reveal the Role of Conserved Function in Electric Organ Convergence Across Electric Fishes
Source: Front Genet. 2019 Jul 18;10:664. doi: 10.3389/fgene.2019.00664 (PMC6657706; doi:10.3389/fgene.2019.00664)

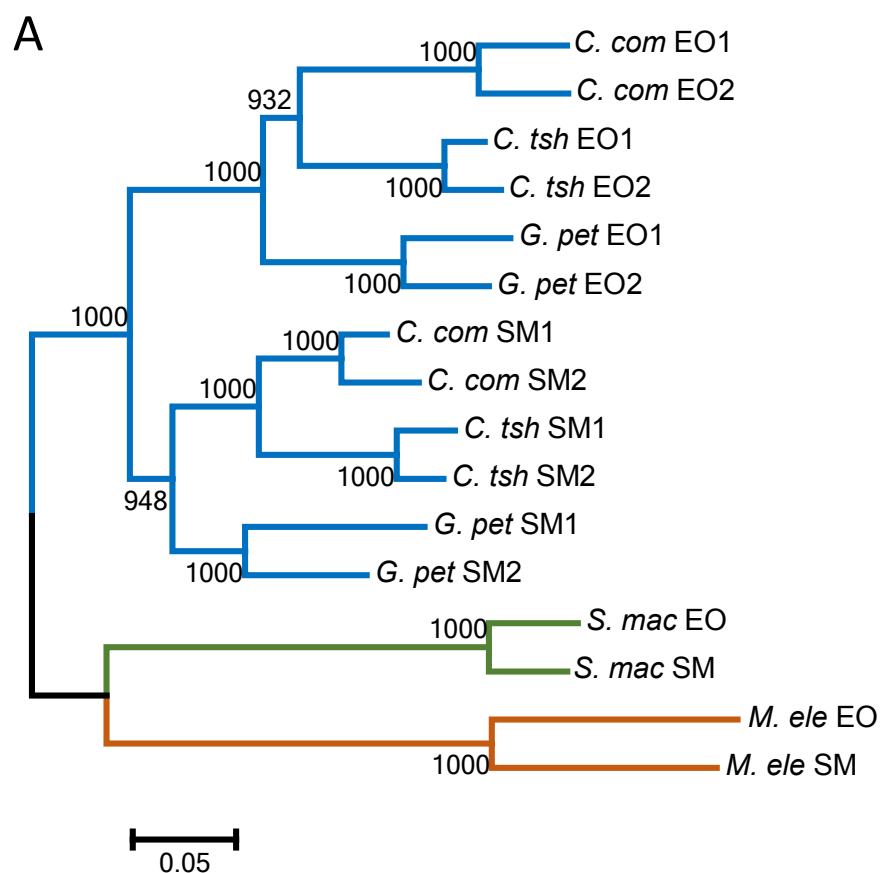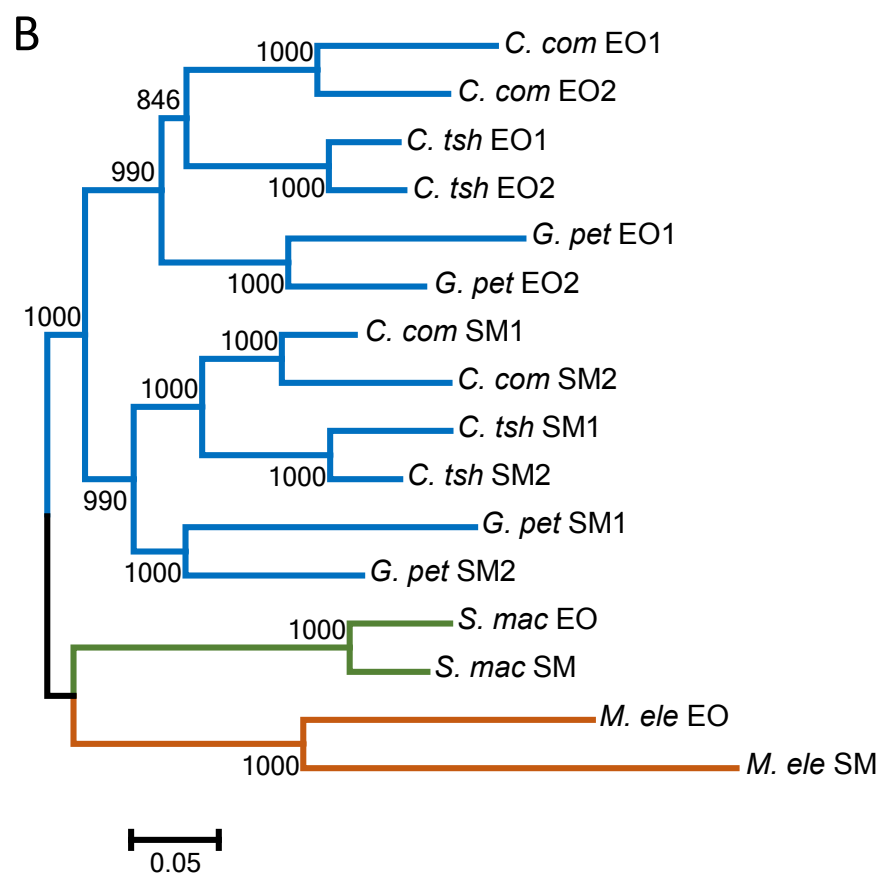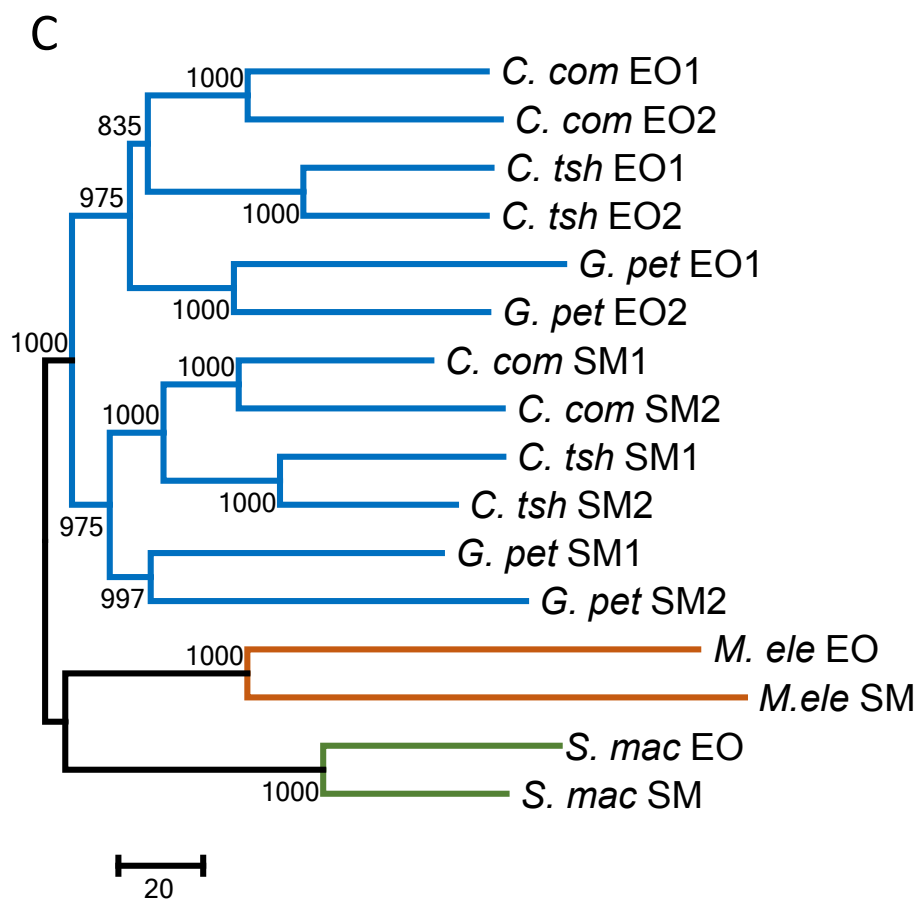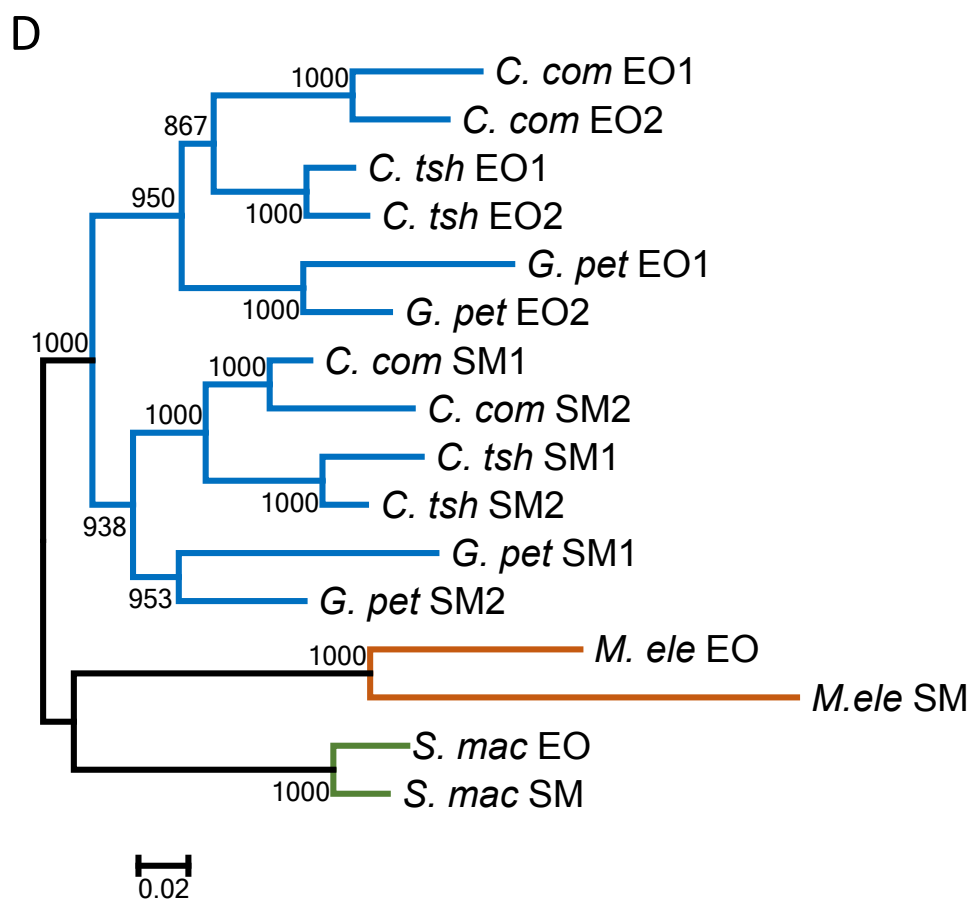

Supplement: Figure S2 — Phylogeny of gene expression based on different vector distances. A-D were constructed based on Pearson distance, Jaccard distance, Euclidean distance, and angular cosine distance, respectively. [file Image_2.pdf]

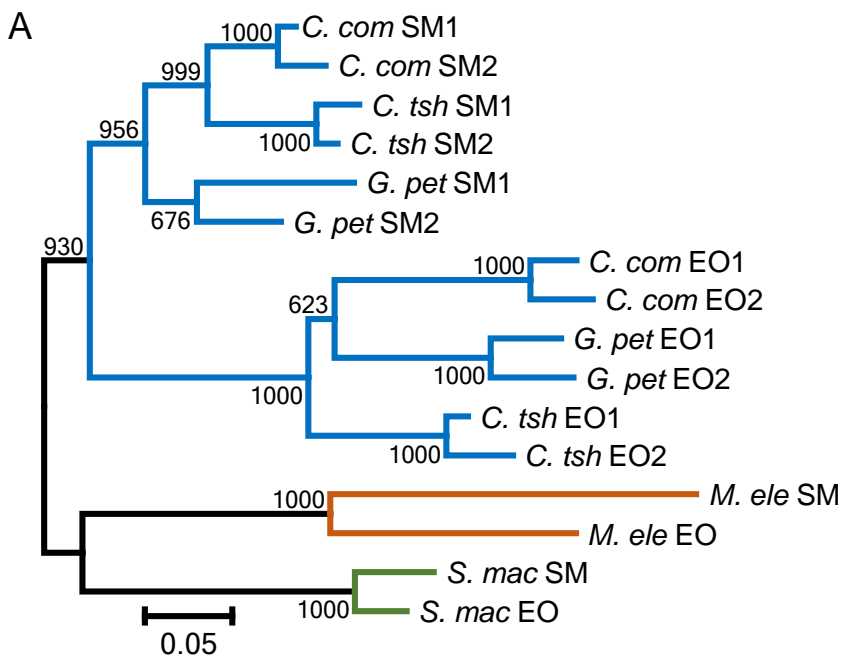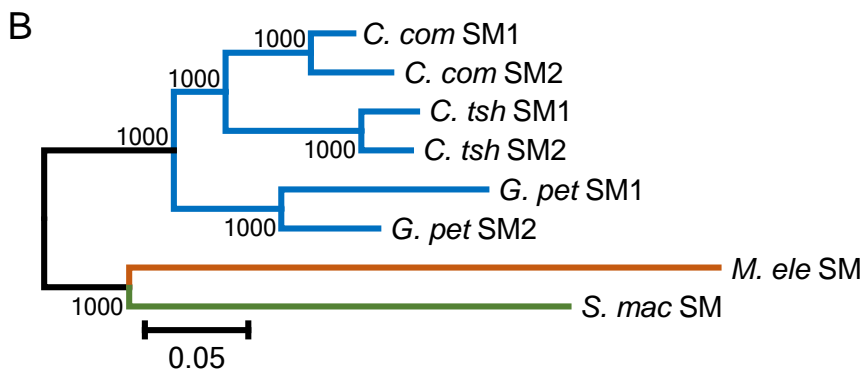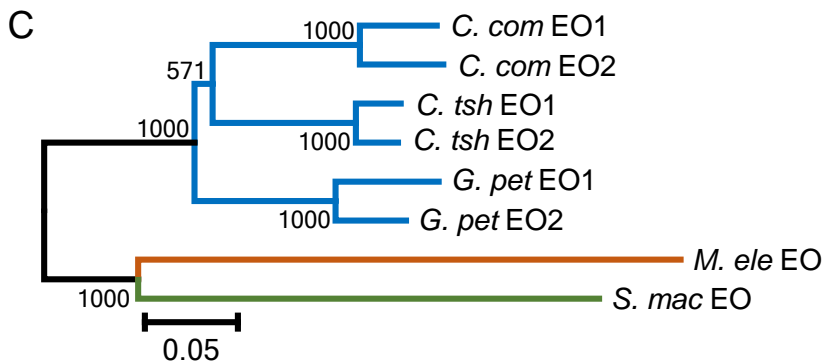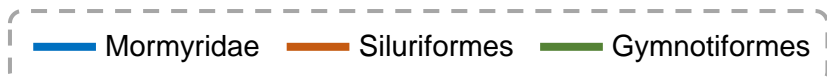

Supplement: Figure S3 — Expression phylogeny of functionally related genes and the EOs and SMs. A. The NJ analysis based on 696 functionally associated genes. The NJ trees of gene expression phylogenies for the SM (B) and the EO (C). The tree topologies were calculated based on pairwise distance matrices (1-ρ). [file Image_3.pdf]

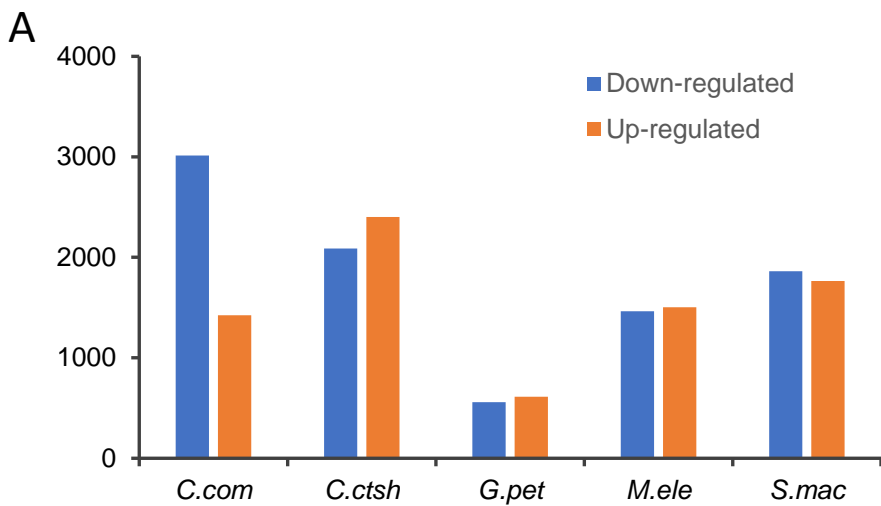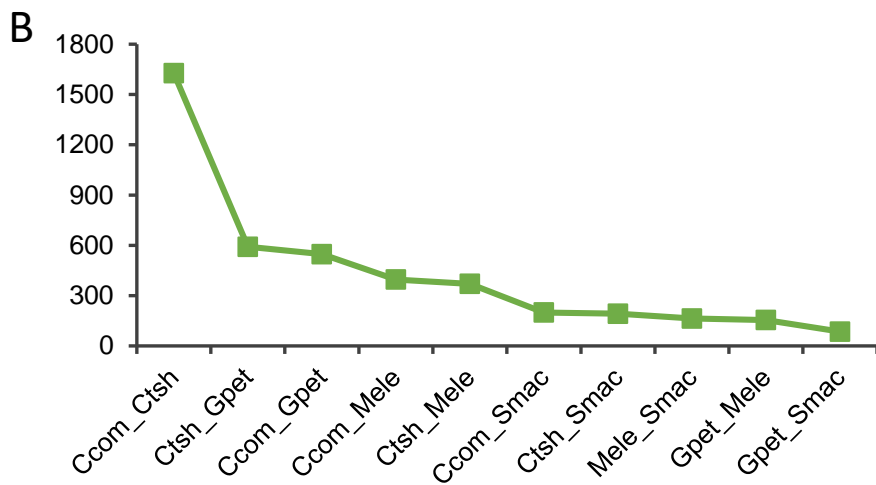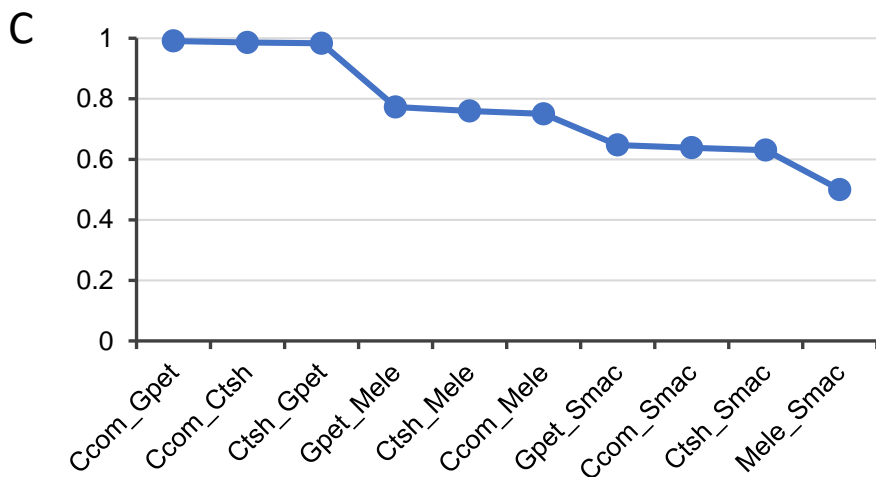

Supplement: Figure S6 — Comparative analysis of DEGs among five electric fishes. [file Image_6.pdf]

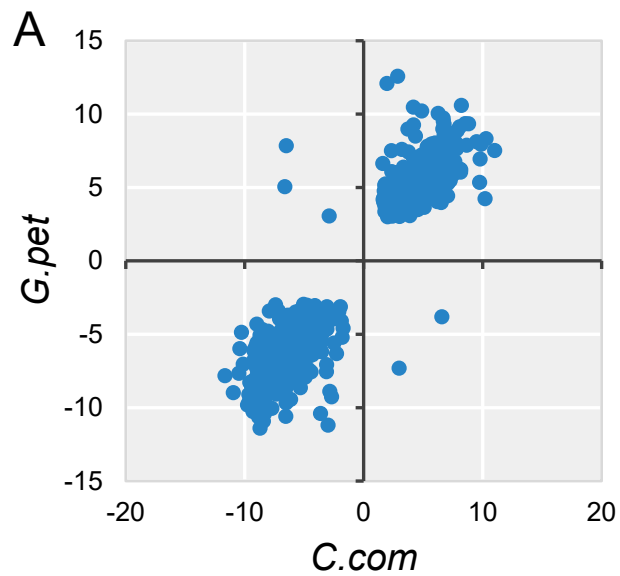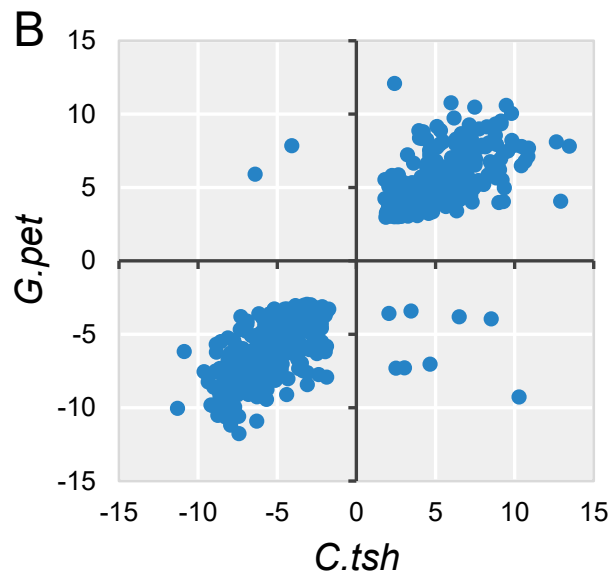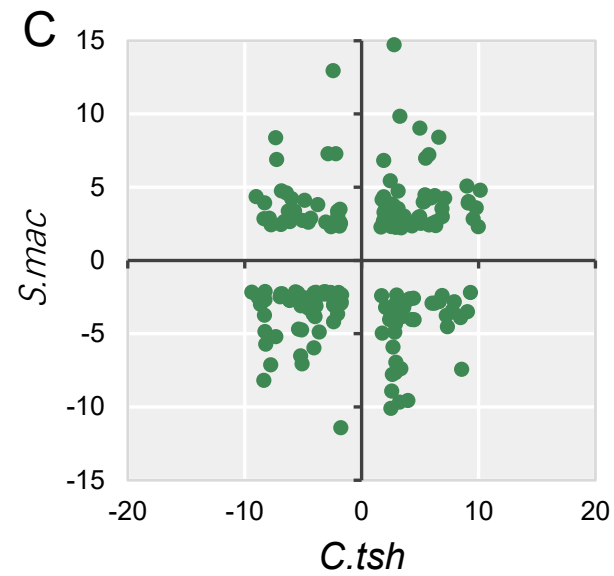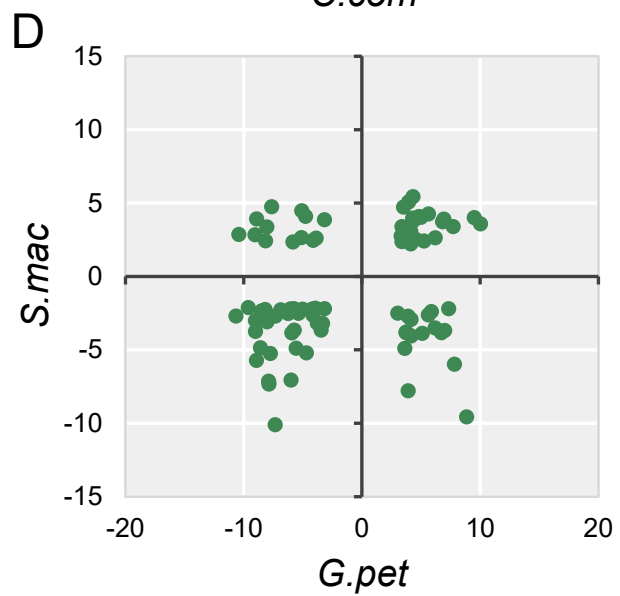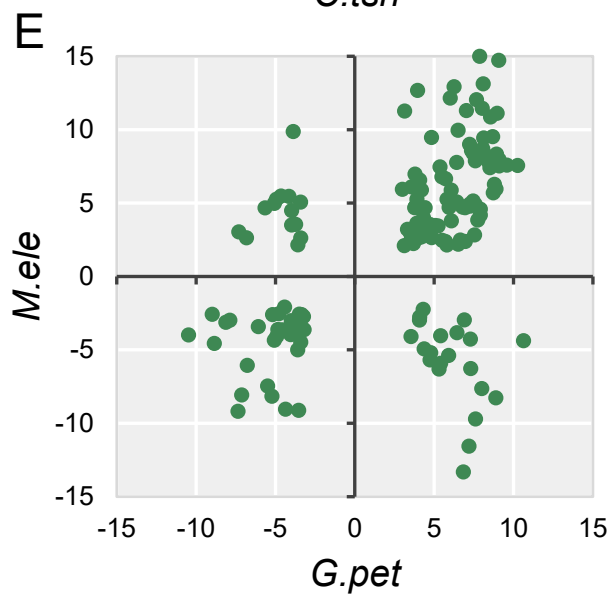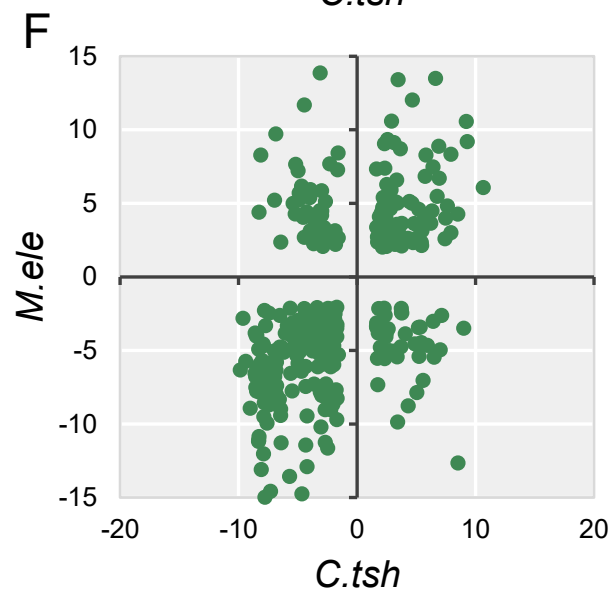

Supplement: Figure S7 — Log2 fold expression change correlation between different species. [file Image_7.pdf]
