# Supplementary material for: Comparative Transcriptome Analyses Reveal the Role of Conserved Function in Electric Organ Convergence Across Electric Fishes
Source: Front Genet. 2019 Jul 18;10:664. doi: 10.3389/fgene.2019.00664 (PMC6657706; doi:10.3389/fgene.2019.00664)
Supplement: Figure S4 — Co-expression and clustering of the EO and SM in electric fish. The gene number of each gene cluster is shown in the lower left corner. The white polyline represents the median centralized expression value of a single gene log2 in each sample, and the red polyline represents the median value of the gene cluster in different samples. The background colors of blue, green, and red represent three species of Mormyroidea, Siluriformes, and Gymnotiformes, respectively. The left part is the SM, and the right part is the EO. [file Image_4.pdf]

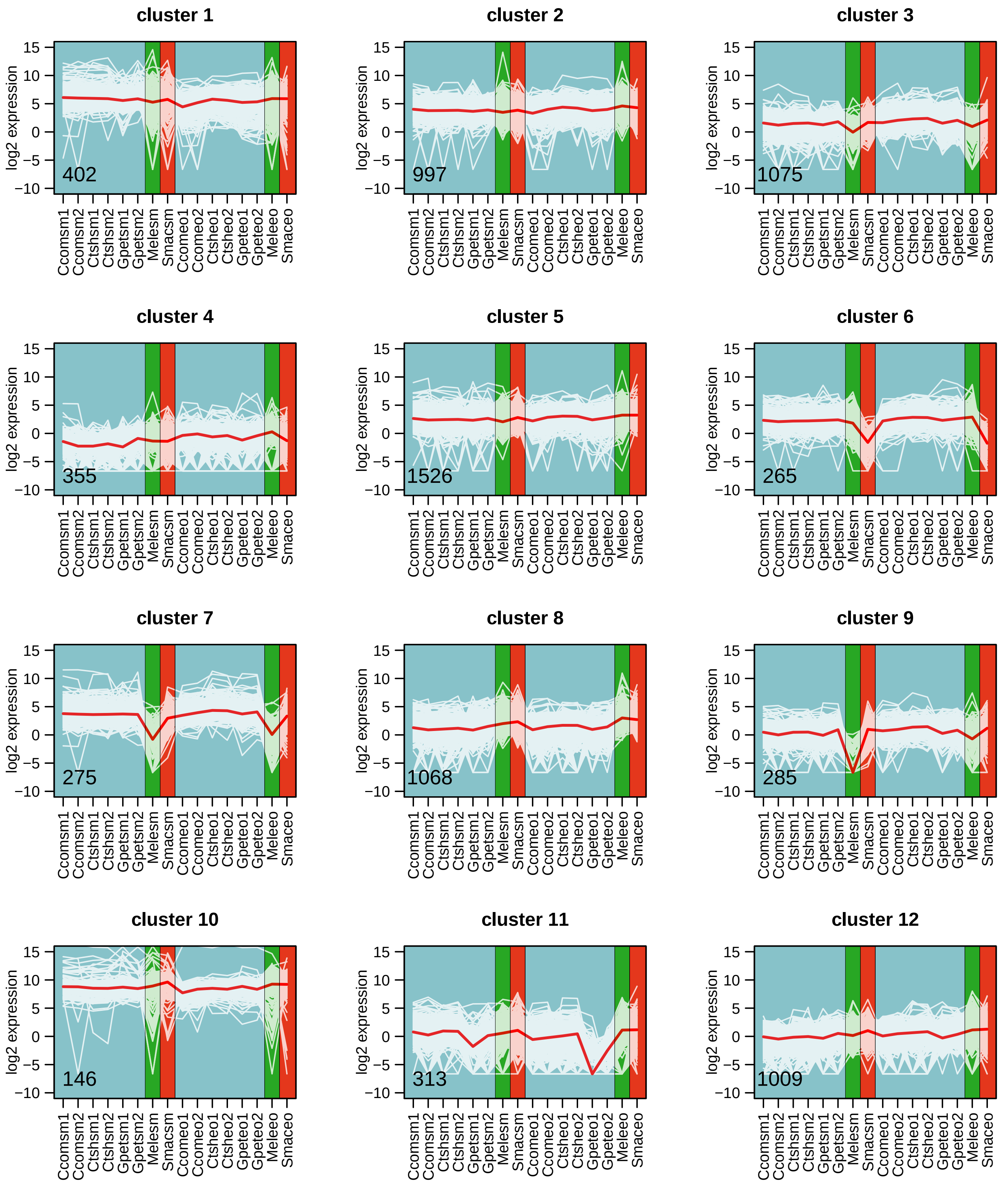
cluster 1
402
cluster 2
997
cluster 3
1075
cluster 4
355
cluster 5
1526
cluster 6
265
cluster 7
275
cluster 8
1068
cluster 9
285
cluster 10
146
cluster 11
313
cluster 12
1009
log2 expression
15
10
5
0
−5
−10
Ccomsm1
Ccomsm2
Ctshsm1
Ctshsm2
Gpetsm1
Gpetsm2
Melesm
Smacsm
Ccomeo1
Ccomeo2
Ctsheo1
Ctsheo2
Gpeteo1
Gpeteo2
Meleeo
Smaceo
