# Supplementary material for: Comparative Transcriptome Analyses Reveal the Role of Conserved Function in Electric Organ Convergence Across Electric Fishes
Source: Front Genet. 2019 Jul 18;10:664. doi: 10.3389/fgene.2019.00664 (PMC6657706; doi:10.3389/fgene.2019.00664)
Supplement: Figure S5 — Comparative analysis of Spearman’s correlation coefficient between 12 expression clusters in different tissues. EO, SM, and E/S represent the correlation coefficients between any two expression profiles of the EOs, SMs, and between EO and SM, respectively. * denotes p < 0.05; ** p < 0.01; *** p < 0.001. [file Image_5.pdf]

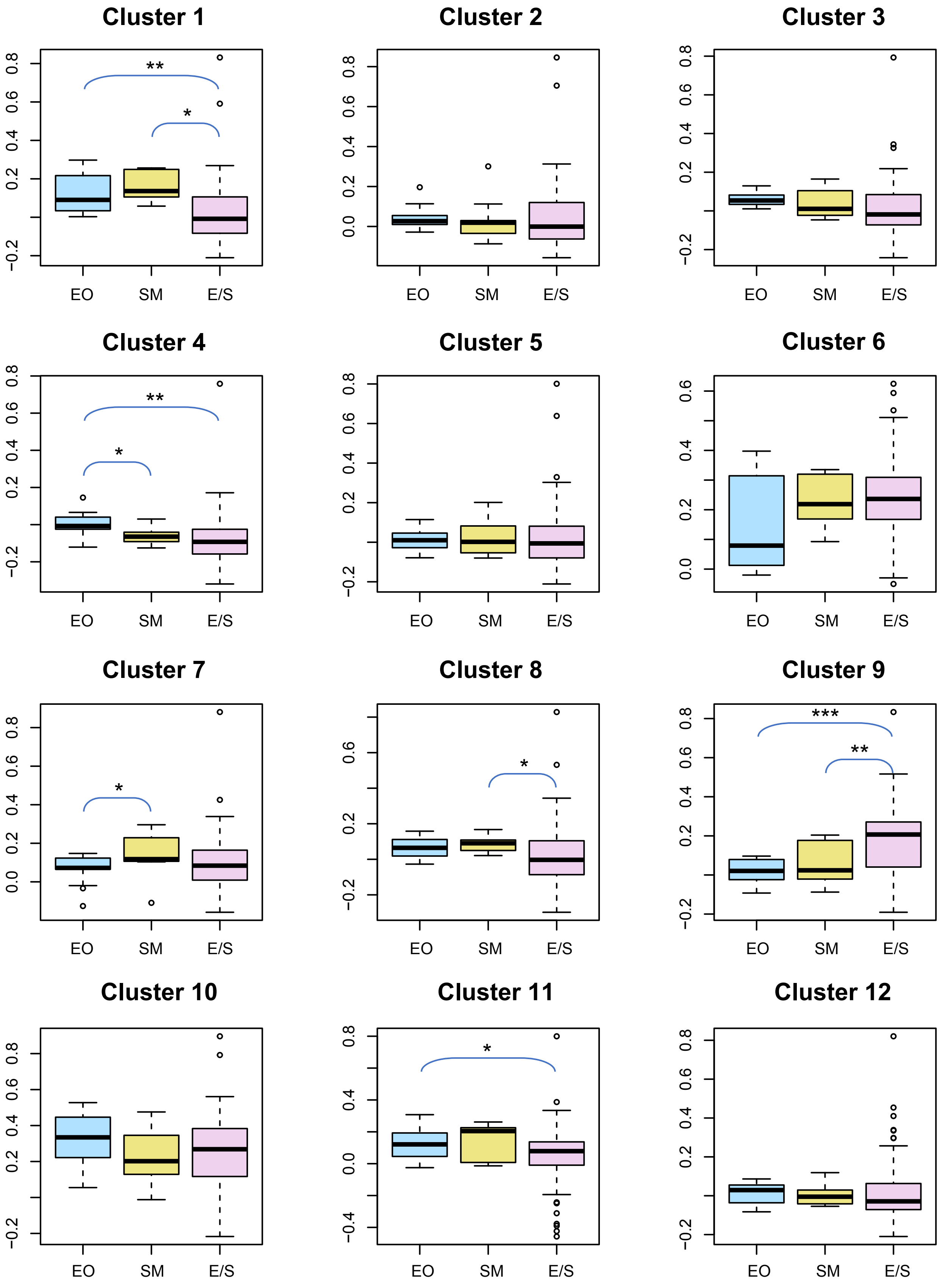
Cluster 1
Cluster 2
Cluster 3
Cluster 4
Cluster 5
Cluster 6
Cluster 7
Cluster 8
Cluster 9
Cluster 10
Cluster 11
Cluster 12
EO
SM
E/S
**
*
***
