# Supplementary material for: Comparative Transcriptome Analyses Reveal the Role of Conserved Function in Electric Organ Convergence Across Electric Fishes
Source: Front Genet. 2019 Jul 18;10:664. doi: 10.3389/fgene.2019.00664 (PMC6657706; doi:10.3389/fgene.2019.00664)
Supplement: Table S1 — The detailed information of raw data used for this study. [file Table_1.docx]

Table S1. The detailed information of raw data used for this study.

| **Lineage** | **Species name** | **Tissue** | **SRA Accession NO.** | **Raw Reads** | **Clean Reads** |
| --- | --- | --- | --- | --- | --- |
| Mormyroidea | *Campylomormyrus compressirostris* | EO | SRX766224 | 17,847,585 | 14,256,543 |
|  |  |  | SRX767393 | 15,645,817 | 12,549,085 |
|  |  | SM | SRX767403 | 37,256,680 | 30,432,777 |
|  |  |  | SRX767400 | 23,932,197 | 19,745,758 |
|  | *Campylomormyrus tshokwe* | EO | SRX767409 | 21,703,048 | 18,362,438 |
|  |  |  | SRX767415 | 37,294,087 | 30,935,270 |
|  |  | SM | SRX767431 | 18,758,922 | 15,007,139 |
|  |  |  | SRX767436 | 64,419,314 | 53,568,031 |
|  | *Gnathonemus petersii* | EO | SRX767474 | 18,910,601 | 14,949,374 |
|  |  |  | SRX767477 | 30,638,159 | 25,123,290 |
|  |  | SM | SRX767478 | 22,868,677 | 18,523,628 |
|  |  |  | SRX767479 | 20,320,459 | 15,843,548 |
| Siluriformes | *Malapterurus electricus* | EO | SRX553133 | 26,992,334 | 20,244,250 |
|  |  | SM | SRX553134 | 17,216,715 | 12,940,369 |
| Gymnotiformes | *Sternopygus macrurus* | EO | SRX553131 | 175,605,480 | 135,216,219 |
|  |  | SM | SRX553132 | 171,612,181 | 131,917,335 |
