# Supplementary material for: Comparative Transcriptome Analyses Reveal the Role of Conserved Function in Electric Organ Convergence Across Electric Fishes
Source: Front Genet. 2019 Jul 18;10:664. doi: 10.3389/fgene.2019.00664 (PMC6657706; doi:10.3389/fgene.2019.00664)
Supplement: Table S3 — Fisher’s exact test for statistically significant overrepresentations of GO categories and KEGG pathways of shared DEGs among different lineages. [file Table_3.docx]

**Table S3. Fisher’s exact test for statistically significant overrepresentations on GO categories and KEGG pathways of shared DEGs among different lineages**

|  | *C.com* Vs *C.tsh* | *C.com* Vs *G.pet* | *C.com* Vs *M.ele* | *C.com* Vs *S.mac* | *C.tsh* Vs *G.pet* | *C.tsh* Vs *M.ele* | *C.tsh* Vs *S.mac* | *G.pet* Vs *M.ele* | *G.pet* Vs *S.mac* | *M.ele* Vs *S.mac* | All Categories among 5 efishes |
| --- | --- | --- | --- | --- | --- | --- | --- | --- | --- | --- | --- |
| GO Term |  |  |  |  |  |  |  |  |  |  |  |
| Shared Terms | 63 | 43 | 39 | 107 | 49 | 42 | 37 | 37 | 25 | 21 | 21 |
| All Terms | 302 | 285 | 284 | 366 | 90 | 92 | 247 | 60 | 222 | 221 | 388 |
| *P* value | 0.704 | 0.034 | 4.25×10^-06^ | <2.2×10^-16^ | 1.67×10^-14^ | <2.2×10^-16^ | 8.49×10^-16^ | <2.2×10^-16^ | 4.74×10^-11^ | 1.81×10^-6^ | <2.2×10^-16^ |
| KEGG pathway |  |  |  |  |  |  |  |  |  |  |  |
| Shared pathways | 11 | 4 | 7 | 15 | 4 | 7 | 9 | 4 | 4 | 7 | 4 |
| All pathways | 27 | 25 | 25 | 27 | 13 | 13 | 21 | 7 | 17 | 17 | 29 |
| *P* value | 0.073 | 0.314 | 0.001 | 6.81×10^-14^ | 0.094 | 3.44×10^-5^ | 2.59×10^-8^ | 0.001 | 0.001 | 1.57×10^-06^ | 3.02×10^-7^ |
